# Supplementary material for: Predicting Angiogenesis by Endothelial Progenitor Cells Relying on In-Vitro Function Assays and VEGFR-2 Expression Levels
Source: Biomolecules. 2019 Nov 8;9(11):717. doi: 10.3390/biom9110717 (PMC6921061; doi:10.3390/biom9110717)
Supplement: Supplementary file 1 [file biomolecules-09-00717-s001.pdf]

Supplementary File for Biomolecules Journal

Predicting angiogenesis by Endothelial Progenitor Cells relying on in-vitro function assays and KDR expression levels

Nadin Sabbah, Tal Tamari, Rina Elimelech, Ofri Doppelt, Utai Rudich and Hadar Zigdon-Giladi

1. Converting optical density (O.D) to cell number in XTT assay

Cells from donor #9 were seeded at gradual concentrations and incubated overnight; O.D values were measured using cell proliferation kit XTT in triplicate according to blank. The average results of four repeats are summarized (Table S1).

Table S1. Average O.D values of human EPCs.

Number of cells seeded per 100µl	O.D (nm)
5,000	0.058
7,500	0.079
10,000	0.093
12,500	0.119
15,000	0.148

XTT Kit was used to analyze the proliferation of human EPCs. During 72 hours, absorbance was examined every 24 hours in quadruplicate. After calculating mean values of relative O.D., results were converted to number of cells using a standardized calibration curve, and according to the mathematical equation: O.D = 10^{-5}*(number of cells) (Figure S1).

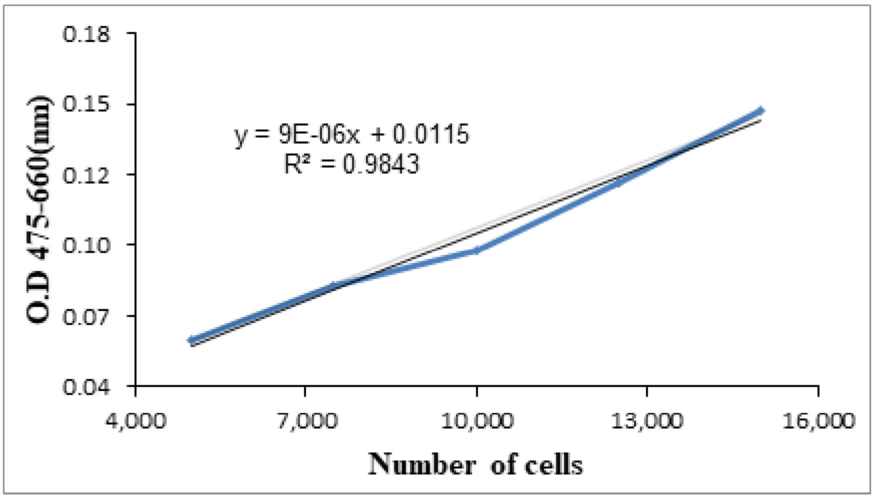

Figure S1. Calibration curve of human EPCs.

According to the calculated values of O.D in Table S1, a straight line equation is represented.

2.A primer list – real-time PCR

Expression levels of angiogenic and chemotactic genes were detected using Real-Time PCR for key genes critical to wound healing and bone repair: SDF1, VEGF-A, CCL2, PDGFβ, KDR, and CXCR4. As an internal control, levels of HPRT were quantified in parallel with target genes (Table S2).

Table S2. Humane primers for Real-Time PCR.

Gene		Forward	Reverse
**Hypoxanthine Phosphoribo-syltransferase 1**	HPRT1	5'-GACCAGTCAACAGGG-GACAT-3'	5'-CCTGACCAAGGAAA-GCAAAG-3'
Stromal cell-derived factor 1	SDF-1	5'-AGAGCCAACGTCAA-GCATCT-3'	5'-CTTTAGCTTCGGGTCAATGC-3'
Vascular endothelial growth factor- A	VEGF-α	5'-TCTTCAA-GCCATCCTGTGTG-3'	5'-TGCATTCACATTTGTT-GTGC-3'
Chemokine (C-C motif) lig-and 2	CCL2	5'-TCTGTGCCTGCTGCTCATAG-3'	5'-GCTTCTTTGGGACACTT-GCT-3'
Platelet-derived growth factor subunit B	PDGFβ	5'-TCCCGAG-GAGCTTTATGAGA-3'	5'-CTCAG-CAATGGTCAGGGAAC-3'
Kinase insert domain receptor (vascular endothelial growth factor receptor 2)	KDR (VEGFR-2)	5'-GCCATGTGGTCTCTCTGGTT-3'	5'-TTCCTCCAACTGCCAA-TACC-3'
C-X-C chemokine receptor type 4	CXCR-4	5'-TCTTTGCCAACGTCAG-TGAG-3'	5'-TGGAGTGTGACAGCTT-GGAG-3'

2. Comparison between donors with high vs. low performances

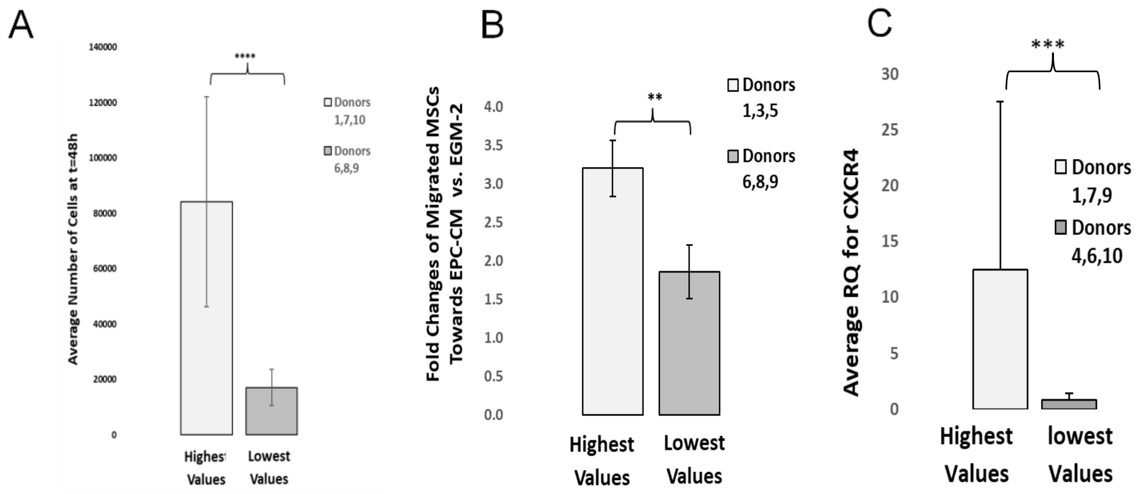

Figure S2. Comparison between donors with high vs. low performances. (**A**) Donors with high vs. low proliferation rate show significant difference, ****$p < 0.0001$. (**B**) Donors with high vs. low chemoattractant ability of EPC-CM show significant difference, **$p < 0.01$. (**C**)Donors with high vs. low expression levels of CXCR4 show significant difference, ***$p < 0.001$.

2. Function and Genotype is NOT Affected by Age

To examine whether age of donors influenced the cells' performance, a Pearson correlation test was carried out. The average result of each *in-vitro* assay for each individual was correlated with the age of the donor. Age-dependent dot plot diagrams were generated for each tested variable: proliferation, migration, and gene expression.

Unexpectedly, the proliferation potential of EPCs that was assessed by the number of cells after 48 and 72 hours did not correlate with the donors' age (Figure S3.A: p = 0.7, R = 0.14 and S3.B: p = 0.09, R = 0.56). High proliferation capacity for more than 80,000 cells after 72 hours was detected in donors over 30 years old, while a lower proliferation was found in donors 23-37 years old (Figure S3.B).

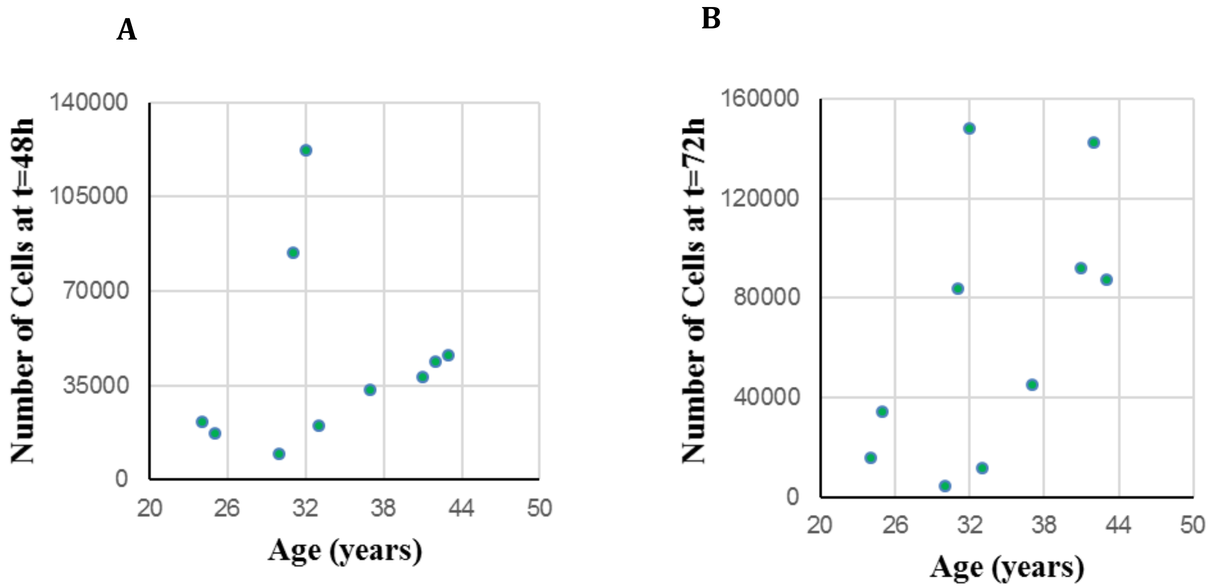

Figure S3. Correlation between donor's age and the number of cells after 48 (**A**) and 72 (**B**) hours.

To verify whether the chemoatractive ability of EPCs' secretome might be affected by human age, the average number of migrated MSC toward EPCs condition media from a specific donor were correlated to the age of that donor. According to Pearson correlation test, no correlations were found between the age of healthy donors and their chemotaxis effect on MSCs ($p = 0.4$, R = 0.3) (Figure S4).

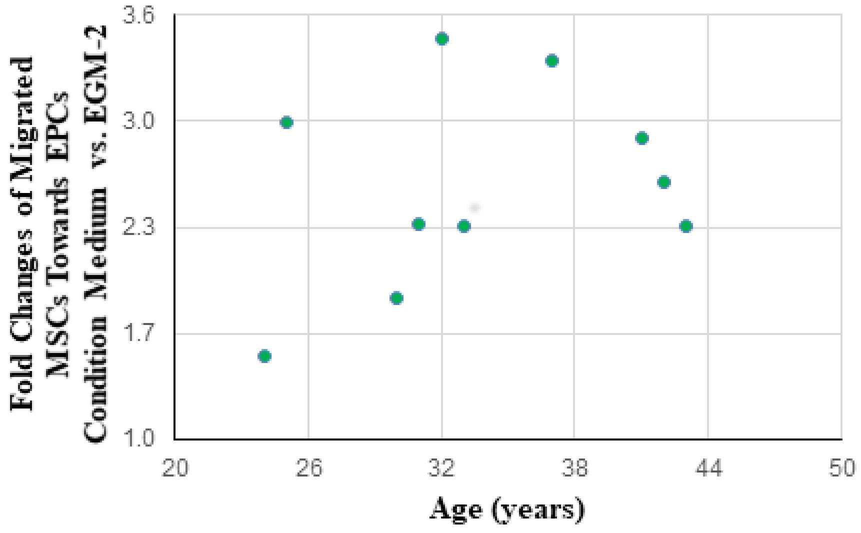

Figure S4. Correlation between donor's age and the chemoatractivy of EPCs concentrated condition medium.

EPC gene expression levels were not correlated with the age of the donor (Figure S5). No correlation was found between healthy donors' age and the expression levels of our gene of interest (Figure S5.A: $p = 0.6$, R = −0.18, S5.B: $p = 0.6$, R = −0.16, S5.C: $p = 0.2$, R = −0.42, S5.D: $p = 0.5$, R = −0.22, S5.E: $p = 0.7$, R = 0.12 and S5.F: $p = 0.8$, R = −0.07).

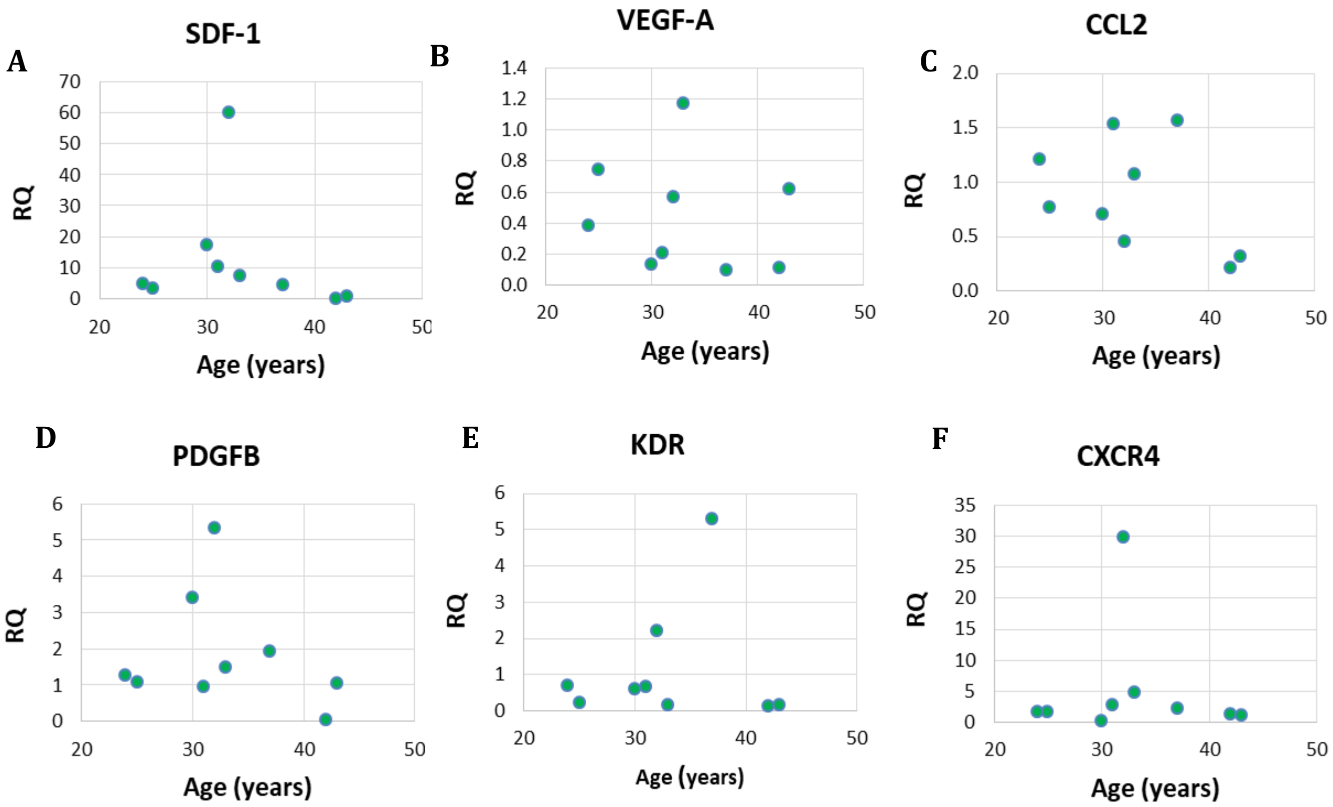

Figure S5. Correlation between donor's age and mRNA levels of angiogenic, osteogenic, and chemotactic genes.

In summary, we were surprised to find no significant correlation between donor's age and EPCs proliferation, chemotactic activity, or gene expression levels.

5. EPCs' Function and Genotype is NOT Affected by Gender

In order to evaluate the effect of patient's gender on cell function and genotype, the results of *in-vitro* assays were dichotomized to two groups according to gender. Wilcoxon-Mann-Whitney test was used to compare the groups.

According to the results, there was no significant difference in EPCs number after 48 hours of cell proliferation assay between females, (mean = 40008.3 ± 26874.4 cells) or males (mean = 47285.0 ± 44305.3 cells, $p = 0.5$). Cell growth rates at 72h were not different between female and male donors (females mean = 64441.7 ± 50084.0 cells vs. males mean = 68716.7 ± 60378.8 cells, $p = 1$), (Figure S6).

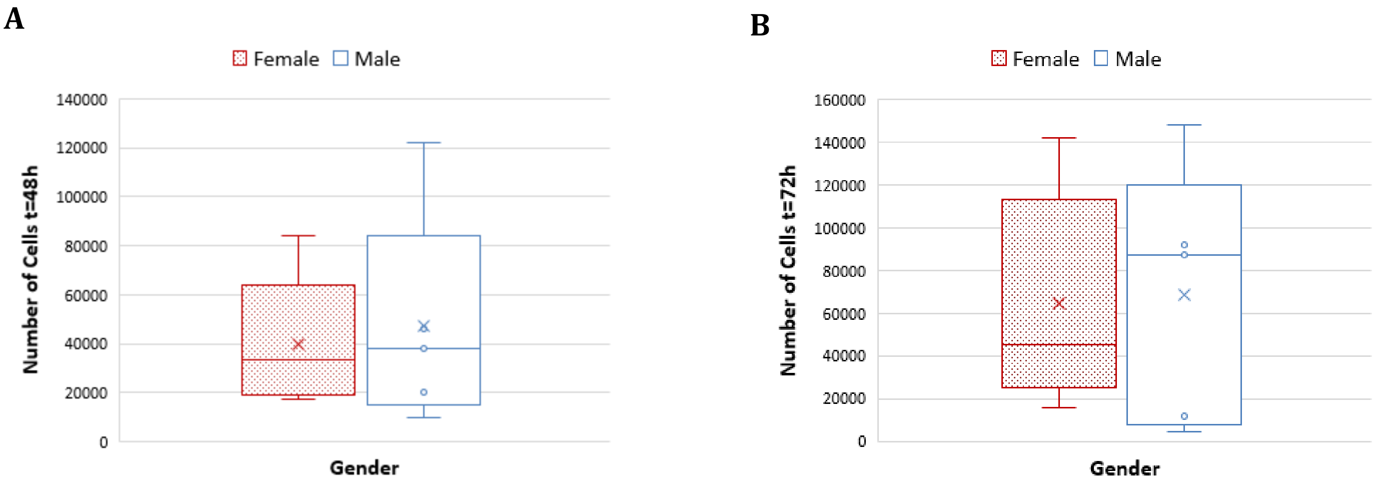

Figure S6. Comparing females and males for cell growth rate.

The chemotactic activity of EPCs secretome was also not affected by gender of the donor. In both groups, MSCs migration toward EPCs concentrated condition media was intensified compared to migration toward EGM-2 (medium without EPC supernatant). As shown in figure S7, both females and males show similar chemotaxis effect on MSCs, as their minimum, median, and maximum values fall into the same values. Correspondingly, no significant difference was noted between the two sub-populations, $p = 0.8$.

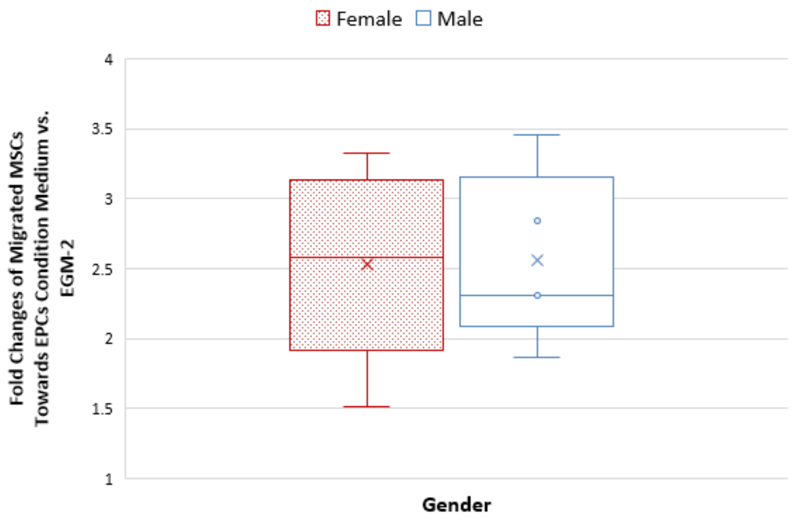

Figure S7. Comparing females and males for chemoatractive activity.

Similar to proliferation and chemoattractant ability, the expression levels of the investigated genes were not affected by the gender of the donor (Figure S8).

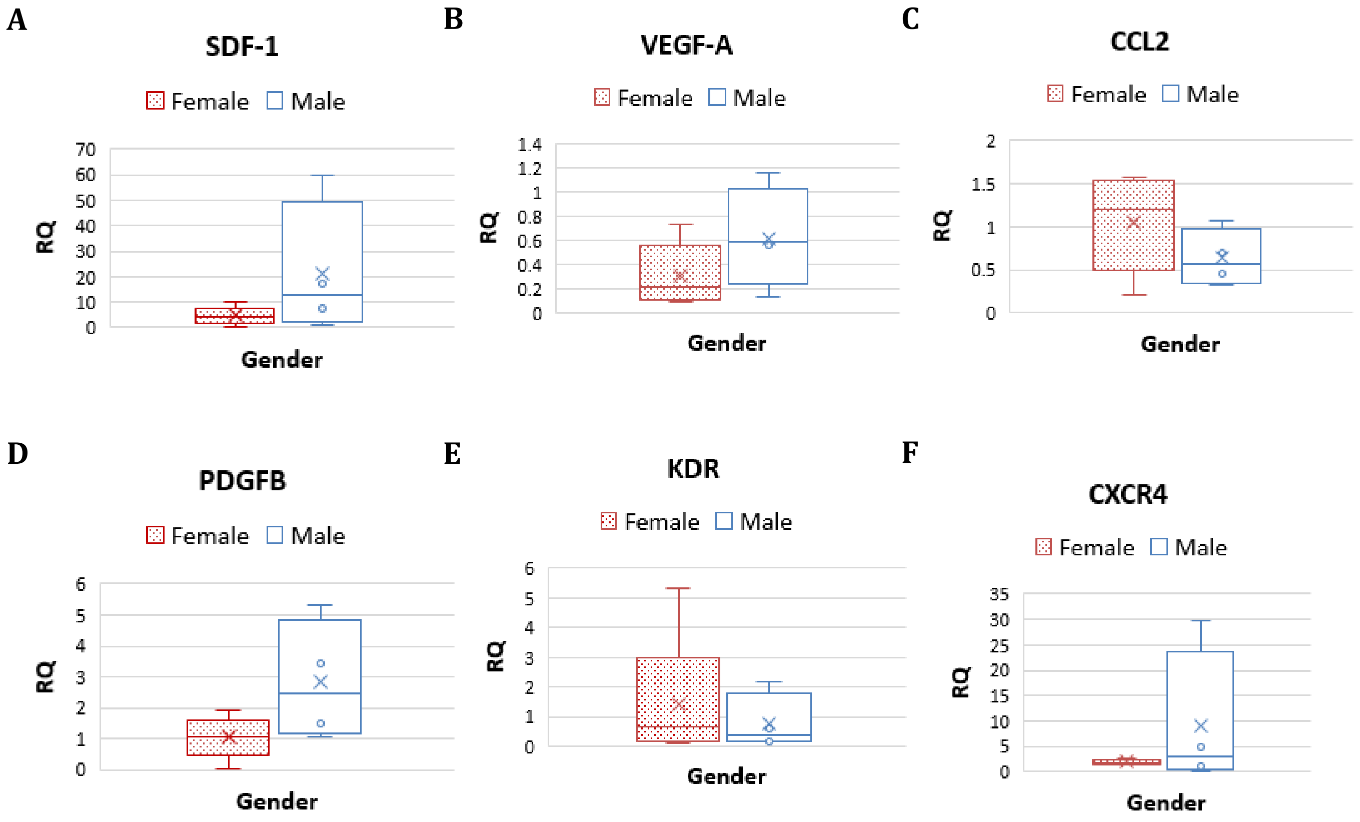

Figure S8. Comparison between female and male for gene expression levels.
